# Radical resection of advanced gallbladder cancer after gemcitabine combined with S-1 and Sintilimab chemotherapy: A case report

**DOI:** 10.1097/MD.0000000000046685

**Published:** 2025-12-19

**Authors:** Jiangwei Mou, Hao Xu, Bo Zhang, Ye Zheng, Sheng Wu, Kexiang Zhu

**Affiliations:** a The First Clinical Medical College, Lanzhou University, Lanzhou, Gansu, P.R. China; b Department of Hepatobiliary Surgery, The First Affiliated Hospital of Zhejiang Chinese Medical University, Zhejiang Provincial Hospital of Chinese Medicine, Hangzhou, Zhejiang, P.R. China; c The First School of Clinical Medicine, Zhejiang Chinese Medical University, Hangzhou, Zhejiang, P.R. China; d Department of General Surgery, The First Hospital of Lanzhou University, Lanzhou, Gansu, P.R. China.

**Keywords:** advanced gallbladder cancer, case report, Immune checkpoint inhibitors, neoadjuvant chemotherapy, sintilimab

## Abstract

**Rationale:**

Gallbladder cancer (GBC) is known for its high malignancy potential, poor patient prognosis, and frequent diagnosis at advanced stages, resulting in a 5-year survival rate of only 5%. For patients with advanced disease who are no longer candidates for curative surgery, preoperative neoadjuvant chemotherapy combined with immune checkpoint inhibitors can help downstage the cancer. This approach may increase the likelihood of successful surgical resection and ultimately improve patient outcomes.

**Patient concerns:**

A 68-year-old female patient presented with intermittent upper abdominal discomfort 1 month ago, without a clear underlying cause.

**Diagnoses:**

The patient has been diagnosed with advanced stage IVB GBC.

**Interventions:**

The patient received 2 cycles of neoadjuvant therapy prior to surgery. The first cycle included a combination of Tegafur, Gimeracil, and Oteracil Potassium (S-1) plus gemcitabine (GS) and sintilimab. Unfortunately, the second cycle of treatment with S-1 was discontinued due to the development of grade III bone marrow suppression. As a result, the treatment was adjusted to gemcitabine in combination with sintilimab. The neoadjuvant chemotherapy effectively reduced the tumor stage. After assessment by a multidisciplinary team (MDT), the patient underwent radical surgery for GBC. Following the surgical procedure, the patient received 2 cycles of gemcitabine monotherapy as adjuvant chemotherapy.

**Outcomes:**

Following the surgery, which occurred 40 months ago, the patient has been receiving regular follow-up examinations at the oncology department. They are in good general condition, with no signs of recurrence or metastasis of the tumor.

**Lessons:**

This case illustrates that combining neoadjuvant therapy with immune checkpoint inhibitors is a crucial approach for enhancing the prognosis of patients with advanced GBC. This strategy increases the likelihood of achieving radical surgical resection by facilitating downstaging treatment, which could enable conversion surgery for patients initially classified as inoperable. In addition, this case successfully demonstrates that sintilimab is a viable, effective, and economically advantageous option for the treatment of advanced GBC transformation.

## 1. Introduction

Gallbladder cancer (GBC) is the most common malignant tumor of the biliary tract.^[1]^ It typically begins without symptoms and can metastasize rapidly in the early stages. As a result, most cases are diagnosed at an advanced stage,^[2]^ leading to a 5-year survival rate of only 5%.^[3]^ Currently, surgical resection remains the only potential cure.^[4]^ However, fewer than 20% of patients are eligible for radical surgery at the time of diagnosis due to local invasion or distant metastasis.^[2]^ In this context, neoadjuvant therapy has emerged as a crucial strategy for improving outcomes in advanced patients. This approach aims to reduce the disease stage to facilitate curative resection and create opportunities for surgery in initially unresectable patients. This case report describes a patient with advanced GBC that was initially unresectable due to involvement of the portal vein, hepatic artery, and retroperitoneal lymph node metastasis. The patient underwent neoadjuvant therapy using a combination of Tegafur, Gimeracil, and Oteracil Potassium (S-1) plus gemcitabine (GS) and the programmed cell death protein 1 (PD-1) inhibitor sintilimab. Following treatment, the tumor significantly shrank, allowing for successful radical resection, with no evidence of lymph node metastasis post-surgery. This case study confirms that the combination of sintilimab and the GS regimen significantly enhances antitumour response, consistent with the findings of Phase III studies such as TOPAZ-1^[5]^ and KEYNOTE-966,^[6]^ which demonstrated that immunotherapy combined with chemotherapy can prolong median overall survival (mOS) and improve objective response rate in patients with advanced biliary tract cancer (BTC). In addition, this case effectively illustrates that a combination treatment regimen including sintilimab is a viable, effective, and affordable option for patients with advanced GBC. Although this is a single case report, the positive outcomes achieved offer strong preliminary evidence and a practical rationale for larger-scale clinical studies. This suggests promising prospects for the broader application of immunotherapy in this area.

### 1.1. Case presentation

The patient is a 68-year-old woman who was admitted to the hospital on February 13, 2022, after experiencing intermittent upper abdominal pain and discomfort for 1 month. Upon admission, the patient’s vital signs were stable, and a physical examination revealed no significant abnormalities aside from tenderness in the upper abdomen. Laboratory tests revealed some abnormal results: Serum carcinoembryonic antigen (CEA): 27.9 ng/mL (normal range: 0–5.2 ng/mL), carbohydrate antigen 12-5 (CA12-5): 54.5 U/mL (normal range: 0–35.0 U/mL), carbohydrate antigen 19-9 (CA19-9):122.0 U/mL (normal range: 0–35.0 U/mL), carbohydrate antigen 24-2 (CA24-2): 26.78 U/mL (normal range: 0–10.0 U/mL), and carbohydrate antigen 72-4 (CA72-4):16.5 U/mL (normal range: 0–6.9 U/mL). Other laboratory results, including complete blood count, albumin, aspartate aminotransferase, alanine aminotransferase, alkaline phosphatase, and alpha-fetoprotein, were within normal limits.

The patient had a 10-year history of hypertension and type 2 diabetes. Her blood pressure and blood glucose levels were well controlled during weekdays. Upon admission, his general condition was fair, and he did not exhibit signs of cachexia. Additionally, there was no reported family history of related genetic conditions.

**Figure 1.**
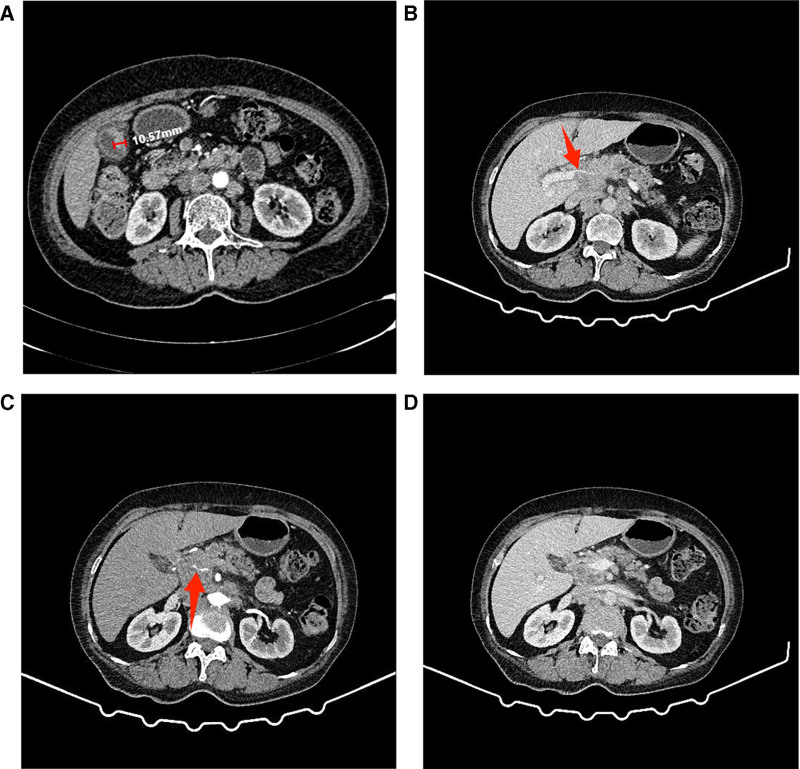
Initial CT findings. (A) Irregular focal wall thickening at the gallbladder fundus, measuring approximately 10.57 mm in thickness. (B) Invasion of the portal vein (indicated by a red arrow). (C) Invasion of the hepatic artery (indicated by a red arrow). (D) Multiple enlarged lymph nodes in the porta hepatis and retroperitoneum.

**Figure 2.**
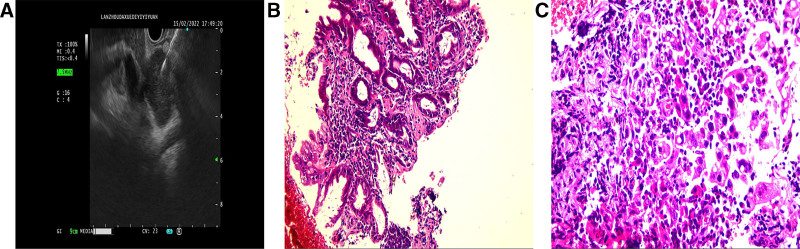
(A) Endoscopic ultrasound-guided fine needle aspiration biopsy of the lymph node at the hepatic hilum. (B) Histological examination of the biopsy from the hepatic hilum lymph node (HE staining, ×100 magnification). (C) Histological examination of the biopsy from the hepatic hilum lymph node (HE staining, ×200 magnification). Both (B and C) confirmed the presence of adenocarcinoma.

Enhanced computed tomography (CT) of the abdomen upon admission revealed uneven thickening and enhancement of the gallbladder’s distal wall (Fig. 1A), invasion of the portal vein (Fig. 1B) and hepatic artery (Fig. 1C), and multiple enlarged lymph nodes in the hepatic hilum and retroperitoneum (Fig. 1D). The diagnosis was GBC with metastases to the lymph nodes in the hepatic hilum and retroperitoneum. An ultrasound-guided endoscopic biopsy of the hepatic hilum confirmed the presence of adenocarcinoma (Fig. 2A–C). After discussions with a multidisciplinary team (MDT), a diagnosis of advanced stage IV B GBC was established. With the consent of the patient and their family, the patient was transferred to our hospital’s Oncology Department on February 17, 2022. After thorough evaluation, it was determined that the patient would receive a chemotherapy regimen consisting of GS combined with sintilimab. The dosing regimen is as follows: on day 1, administer 1400 mg of gemcitabine (Qilu Pharmaceutical Co., Ltd., Haikou City, Hainan Province, China) and 200 mg of sintilimab (Innovent Biologics Co., Ltd., Suzhou, Jiangsu Province, China) via intravenous infusion. On day 8, administer an additional 1400 mg of gemcitabine by intravenous infusion. From days 1 to 14, the patient will take 60 mg of S-1 (Qilu Pharmaceutical Co., Ltd., Jinan City, Shandong Province, China) orally twice daily. During the first cycle of chemotherapy, a routine blood test on day 6 revealed myelosuppression. The laboratory findings were as follows: a white blood cell count of 1.77 × 10⁹/L (reference range, 4–10 × 10⁹/L), an absolute neutrophil count of 1.25 × 10⁹/L (reference range, 2–7 × 10⁹/L), and a red blood cell count of 2.71 × 10¹²/L (reference range, 3.5–5.0 × 10¹²/L). These findings were consistent with a diagnosis of grade Ⅲ myelosuppression. Consequently, the oral administration of S-1 was immediately discontinued, and Leucogen Tablets with Caffeic Acid Tablets were administered orally to elevate blood cell counts. After 4 days of intervention, a repeat complete blood count showed that all parameters had returned to the normal range. To prevent the recurrence of myelosuppression in subsequent chemotherapy cycles, a subcutaneous injection of Pegylated recombinant human granulocyte colony-stimulating factor at a dose of 6 mg was administered prophylactically. Subsequently, the treatment regimen was adjusted to gemcitabine combined with sintilimab for continuation.

**Figure 3.**
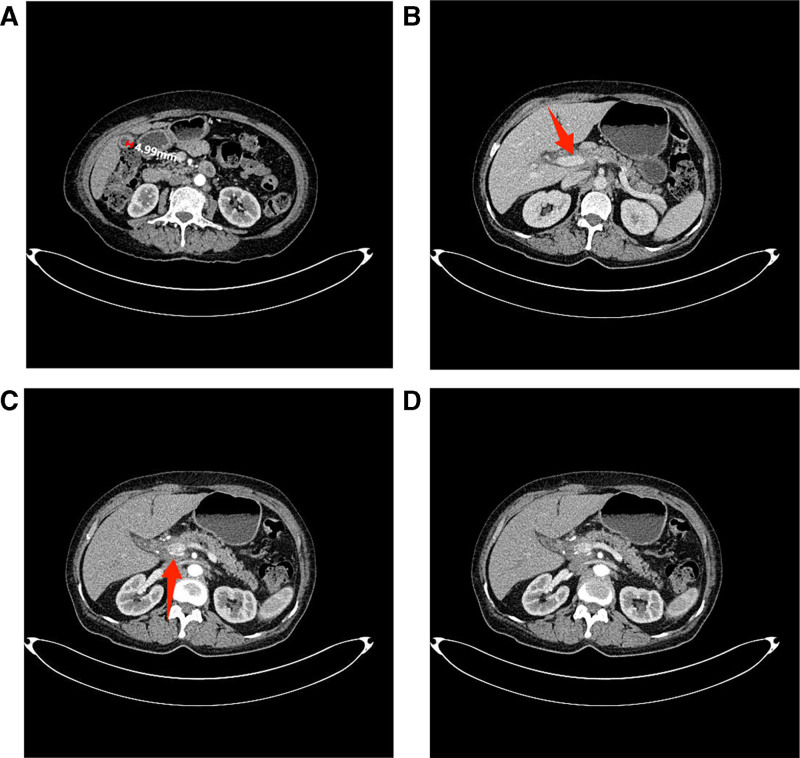
Post-chemotherapy CT findings. (A) The thickness of the gallbladder fundal wall measures approximately 4.99 mm. (B) Marked improvement in portal vein invasion (marked by the red arrow). (C) Marked improvement in hepatic artery invasion (marked by the red arrow). (D) Multiple enlarged lymph nodes in the porta hepatis and retroperitoneum have significantly regressed compared to the pre-chemotherapy scan.

The dosing regimen for the second cycle of chemotherapy is as follows: on the first day, administer 1400 mg of gemcitabine and 200 mg of sintilimab via intravenous infusion. On day 8, administer 1400 mg of gemcitabine by intravenous infusion. The patient’s gastrointestinal reactions and bone marrow suppression were both graded as level I. After 2 cycles of chemotherapy, laboratory tests indicated that, except for CA72-4 (12.1 U/mL), which remained above normal levels, the other tumor markers – CEA, CA12-5, CA19-9, and CA24-2 – had all decreased to within the normal range. Changes in tumor marker levels before and after chemotherapy are presented in Table 1. Enhanced CT scanning of the entire abdomen showed that the thickness of the gallbladder fundal wall decreased from 10.57 mm to 4.99 mm (Fig. 3A), and the invasion of the central portal vein (Fig. 3B) and hepatic artery (Fig. 3C) was notably less severe. Additionally, the multiple enlarged lymph nodes in both the hepatic hilum and retroperitoneum also had significantly decreased in size (Fig. 3D).

**Table 1:** Changes in tumor markers before and after chemotherapy.

Tumour marker	Prior to treatment	Posttreatment	Reference range
CEA	27.9	2.3	0–5.2 (ng/mL)
CA12-5	54.5	18.6	0–35.0 (U/mL)
CA19-9	122.0	16.3	0–35.0 (U/mL)
CA24-2	26.78	8.99	0–10.0 (U/mL)
CA72-4	16.5	12.1	0–6.9 (U/mL)

**Figure 4.**
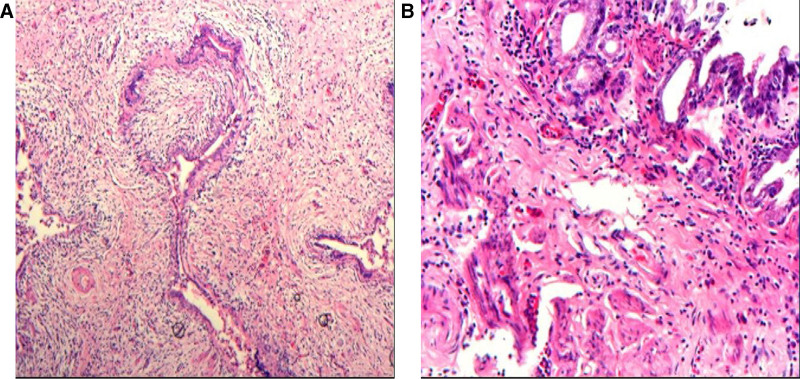
Postoperative pathology: (A) HE staining, ×100 magnification. (B) HE staining, ×200 magnification. (A and B) show a small amount of moderately differentiated adenocarcinoma remaining in the gallbladder, consistent with the changes observed after treatment.

The MDT evaluation indicated significant improvement compared to the patient’s initial condition, leading to a recommendation for radical surgery for GBC. After providing informed consent, the patient underwent a successful radical GBC resection under general anesthesia on April 18, 2022. There were no complications during the procedure, and the patient was discharged on postoperative day 7 following a satisfactory recovery. Postoperative histopathology revealed residual moderately differentiated adenocarcinoma in the gallbladder, consistent with posttreatment changes. The cancerous tissue had invaded the subserosal layer, but all surgical margins were negative for malignant tumors. Additionally, no evidence of metastatic cancer was found in the lymph nodes (8A, 9, 12A, 12P, 13, and 16B). The final pathological staging was ypT2N0M0 (stage IIB) (Fig. 4A and B).

**Figure 5.**
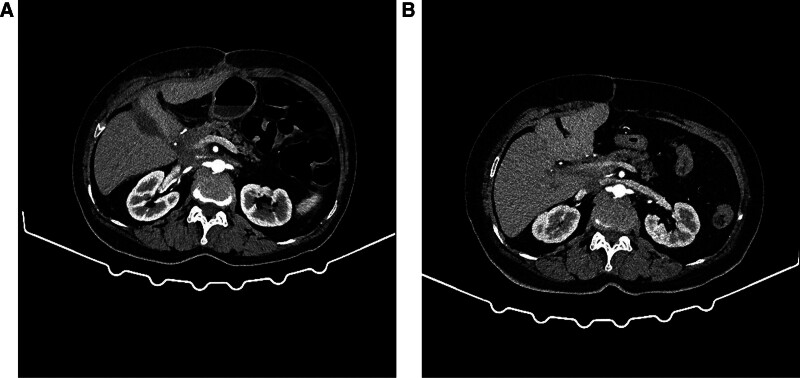
Follow-up abdominal contrast-enhanced CT scans. Both scans demonstrate no evidence of significant abnormalities. (A) One-month postoperative scan. (B) Scan from January 3, 2024. CT = computed tomography.

Under the guidance of the physician, the patient underwent a follow-up abdominal contrast-enhanced CT (Fig. 5A) and continued systemic therapy at our hospital’s Department of Medical Oncology 1 month after surgery. Between May 26 and July 7, 2022, the patient underwent 2 cycles of adjuvant chemotherapy with gemcitabine monotherapy. The dosing regimen for each cycle involved administering 1400 mg of gemcitabine intravenously on Days 1 and 8. Since the surgery, which was 40 months ago, the patient has been undergoing regular follow-up examinations (Fig. 5B) at the Department of Medical Oncology and remains in good general health, with no signs of recurrence or metastasis of the tumor.

## 2. Discussion

GBC is primarily classified as an adenocarcinoma. Most patients are diagnosed at an advanced stage, often accompanied by peripheral vascular invasion, which is associated with high malignancy and poor prognosis.^[7]^ When tumors invade the portal vein or hepatic artery, they are typically considered advanced and inoperable.^[4]^ In this particular case, an enhanced CT scan of the entire abdomen revealed invasion of both the portal vein and hepatic artery, along with multiple enlarged lymph nodes in the hepatic hilum and retroperitoneum. As a result, the patient was diagnosed with unresectable advanced GBC.

Neoadjuvant therapy has emerged as a significant treatment option for unresectable advanced malignant tumors. This approach not only has the potential to reduce tumor staging and increase the likelihood of curative surgery but also to prolong patient survival.^[8]^ Neoadjuvant chemotherapy is particularly utilized to shrink tumors and facilitate surgical resection in malignant tumors of the digestive system, such as gastric, pancreatic, and esophageal cancers.^[9]^ Current research is also exploring neoadjuvant therapies for BTC. A retrospective evaluation found that among patients with initially unresectable cholangiocarcinoma, the rate of surgical resectability after receiving neoadjuvant chemotherapy was 39%.^[10]^ For instance, Kato et al^[11]^ reported in 2013 that 8 out of 22 patients with locally advanced BTC who underwent chemotherapy successfully underwent conversion surgery. Similarly, Al Jaber et al^[7]^ documented that 15 patients with advanced GBC, initially deemed inoperable, experienced tumor shrinkage through neoadjuvant chemotherapy, which ultimately enabled surgical resection. The chemotherapy regimens used for these patients included: 5 cases were treated with gemcitabine alone; 1 case with S-1 alone; 2 cases with GS; and 7 cases with gemcitabine plus cisplatin (GC). A phase III clinical trial has confirmed that S-1 chemotherapy has become the standard treatment for biliary tract tumors in Asia.^[12]^ Currently, the primary first-line chemotherapy regimens for GBC consist of either GC or GS. The GCS regimen, which combines gemcitabine, cisplatin, and S-1, is also considered a potential alternative.^[4]^ Studies have shown that, compared with S-1 monotherapy, the GS regimen demonstrated higher overall survival (OS), progression-free survival (PFS), and response rates.^[13]^

The standard first-line systemic treatment for advanced BTC has remained unchanged for a considerable time. Since the ABC-02 trial established the standard regimen of GC chemotherapy, which has a mOS of 11.7 months, researchers have focused on exploring adjuvant strategies over the past decade. Significant advancements have been made in immunotherapy based on immune checkpoint inhibitors (ICIs).^[14]^ ICIs primarily consist of monoclonal antibodies that target key proteins such as cytotoxic T lymphocyte-associated protein 4 (CTLA-4), PD-1, and programmed cell death ligand 1 (PD-L1). The main mechanism of these therapies is to block the interaction between PD-1, mainly present on activated T cells, and PD-L1, which is primarily expressed on tumor cells and some immune cells. This blockade enhances the body’s antitumor immune response.^[15]^ In the case of GBC, the positivity rate of PD-L1 in tumor cells (with a cutoff value set at ≥ 1%) has varied between 14.7% and 23% and has been linked to a poor prognosis.^[16]^ Additionally, The expression levels of PD-1 in advanced biliary tract malignant tumors were not significantly associated with the efficacy of different ICIs or PFS. However, combination strategies such as ICIs combined with chemotherapy or targeted therapy were independently associated with improved PFS.^[17]^ Gou et al^[18]^ found that, compared to chemotherapy alone, PD-1 inhibitors combined with chemotherapy significantly extended PFS in patients with advanced GBC, and the adverse events associated with this combination were manageable. Subsequent studies revealed that mOS was notably higher in the PD-1 inhibitor plus chemotherapy group, with a mOS of 14.9 months compared to 4.1 months for the PD-1 inhibitor monotherapy group and 6.0 months for the chemotherapy monotherapy group. Additionally, Median PFS was also significantly longer in the PD-1 inhibitor plus chemotherapy group (5.1 months) than in the PD-1 inhibitor monotherapy group (2.2 months) or the chemotherapy monotherapy group (2.4 months).^[19]^ The results of the Ⅲ phases of the TOPAZ-1 trial, published in 2022, indicated that adding the PD-L1 inhibitor durvalumab to the standard GC regimen significantly improved survival in previously untreated patients with advanced BTC compared to the placebo group. Specifically, mOS increased from 11.5 months to 12.8 months, and objective response rate increased from 18.7% to 26.7%.^[5]^ Similarly, the KEYNOTE-966 study showed that combining the PD-1 inhibitor pembrolizumab with GC significantly increased mOS (12.7 months vs 10.9 months) and PFS (6.5 months vs 5.6 months) compared to chemotherapy alone.^[6]^ These groundbreaking advances establish the combination of immunotherapy and chemotherapy as the new standard first-line treatment for advanced BTC.

Various types of ICIs are used to treat advanced BTC, including durvalumab, pembrolizumab, sintilimab, and nivolumab.^[14]^ Sintilimab is a fully human, recombinant, IgG4-type anti-PD-1 monoclonal antibody that is effective against both adenocarcinoma and squamous cell carcinoma. It has a higher affinity for human PD-1 than nivolumab and pembrolizumab and shows excellent PD-1 binding affinity and antitumour activity in humanized mouse models.^[20,21]^ Additionally, sintilimab is effectively metabolized and has an acceptable safety profile in vivo, with a serum half-life of 35.6 hours, which is significantly shorter than that of nivolumab (43.5 hours) and pembrolizumab (42.5 hours).^[22]^ In summary, sintilimab possesses broad-spectrum antitumour activity, high affinity, potent antitumour effects, and favorable pharmacokinetics and safety characteristics. In terms of clinical comparisons, Zheng et al^[23]^ reported superior OS values in the durvalumab group compared with the sintilimab group (19.3 months vs 10.2 months). However, due to the limited sample size and baseline heterogeneity of the study, the results cannot conclusively determine the superiority of durvalumab. In addition, economic factors are also an important consideration. Compared to the high cost of durvalumab and other ICIs, sintilimab has a significant price advantage as it is included in China’s medical insurance reimbursement scope. In summary, the GS chemotherapy regimen combined with sintilimab was ultimately chosen for treatment based on the patient’s high PD-1 expression, the characteristics of the drugs, and economic accessibility.

For the initial systemic treatment of advanced GBC, both the GC and GS regimens are recommended standard options according to domestic and international guidelines. Results from the Phase III FUGA-BT (JCOG1113) clinical trial showed that the GS group was noninferior to the GC group in terms of both mOS (15.1 months vs 13.4 months) and PFS (6.8 months vs 5.8 months). Additionally, a comparison between the 2 groups indicated that their objective tumor response rates and overall safety profiles were similar. Moreover, S-1, used in the GS regimen, is an oral formulation, which eliminates the need for intravenous hydration that is required with cisplatin in the GC regimen. This offers advantages in terms of convenience and patient compliance.^[24]^ Therefore, considering the patient’s immunological profile, which is characterized by high PD-1 expression, along with factors like drug accessibility, treatment convenience, and the potential benefits of combination immunotherapy, the GS chemotherapy regimen combined with the PD-1 inhibitor sintilimab was ultimately chosen as the first-line treatment.

At the initial diagnosis, the patient presented with invasion of both the portal vein and hepatic artery, along with multiple enlarged lymph nodes in the hepatic hilum and retroperitoneum. After receiving 2 cycles of neoadjuvant chemotherapy combined with immunotherapy, the patient exhibited a positive response to the treatment. However, the patient experienced grade III bone marrow suppression during the first cycle of GS chemotherapy, leading to the discontinuation of oral S-1. Although the GS regimen could not be fully implemented due to the halt of S-1, the combination of chemotherapy with gemcitabine and the immunotherapy drug sintilimab proved to be significantly effective in this case. After a MDT assessment, the patient underwent conversion surgery, during which a successful radical resection of the tumor was achieved. Postoperative pathology confirmed that all lymph nodes in the examined regions were negative for cancer. Radical resection for GBC is associated with improved survival rates.^[25]^ Compared to locally advanced GBC patients who did not undergo radical surgery, those who had radical surgery following neoadjuvant chemotherapy showed a significantly longer mOS. Furthermore, neoadjuvant chemotherapy may provide benefits for approximately one-third of advanced GBC patients in terms of OS and PFS.^[26]^ This approach may also offer survival advantages for patients with GBC involving the hepatic artery, portal vein, or adjacent organs.^[27]^ Among patients with extrahepatic cholangiocarcinoma, those who underwent surgical resection after neoadjuvant chemotherapy demonstrated improved long-term survival rates.^[28]^ Additionally, patients with lymph node-positive GBC experienced a significantly longer survival period in the neoadjuvant chemotherapy group compared to those who underwent surgery alone (30 months vs 14 months).^[27]^

## 3. Conclusion

The current standard of care for GBC primarily relies on chemotherapy, such as the GC and GS regimens. However, new treatment strategies are emerging, including targeted immunotherapy combinations (e.g., lenvatinib paired with PD-1 inhibitors) and chemoradiotherapy. This case highlights a successful example of individualized treatment for patients with initially unresectable GBC, demonstrating the feasibility of combining sintilimab with chemotherapy, even in settings with limited resources. In the future, it will be important to explore further precision treatment strategies based on biomarkers, such as PD-1 expression levels, to enhance the success rate of translational therapy. This case illustrates that with careful selection of ICIs combined with chemotherapy regimens, along with comprehensive management by a MDT, some patients with initially unresectable advanced GBC can achieve radical surgical resection, significantly improving their prognosis. While the success in a single case does not imply broad applicability, this comprehensive treatment strategy demonstrates the potential to address the current therapeutic limitations in advanced GBC and suggests a new direction for exploration.

## Author contributions

**Conceptualization:** Jiangwei Mou, Kexiang Zhu.

**Investigation:** Ye Zheng.

**Visualization:** Sheng Wu.

**Writing – original draft:** Jiangwei Mou.

**Writing – review & editing:** Hao Xu, Bo Zhang, Kexiang Zhu.
